# Race and racism: are we too comfortable with comfort?

**DOI:** 10.3399/BJGPO.2020.0143

**Published:** 2020-11-25

**Authors:** Dipesh P Gopal, Salman Waqar, Victoria Silverwood, Ebrahim Mulla, Olamide Dada

**Affiliations:** 1 Institute of Population Health Sciences, Barts and The London School of Medicine and Dentistry, London, UK; 2 Nuffield Department of Primary Care Health Sciences, University of Oxford, Oxford, UK; 3 School for Primary, Community and Social Care, Keele University, Keele, UK; 4 Division of Primary Care, University of Nottingham, Nottingham, UK; 5 Cardiff University School of Medicine, UHW Main Building, Cardiff, UK; 6 Melanin Medics, London, UK

**Keywords:** Ethnic groups, racism, anti-racism

## 



## Introduction

After the murder of George Floyd and the Black Lives Matter protests, there has been a renewed appetite to tackle racism in all aspects of life. Within medicine and especially primary care, many people seem unclear about the racial disparities in health contexts, society, and beyond. This editorial highlights how racism and racial disparities can interweave and influence a day in the life of a GP.

### Practising

It can be easy to miss the health disparities between White people and non-White people in the four walls of our consulting rooms and busy appointment lists. It is well known that people from Black, Asian, and minority ethnic (BAME) communities experience poorer health outcomes than their White counterparts. This phenomenon occurs across many different diseases^1^. In some cases its role is well-known, as in diabetes; it is lesser known in other cases such as asthma and cancer care. It’s particularly striking to note a fivefold higher maternal mortality^2^ among Black women compared to White women in the UK. Socioeconomic factors are more likely than genetic factors^3^ to explain why some groups experience more illness than others.

A significant proportion of patients from ethnic minorities struggle to get the healthcare they need,^4^ are afraid to complain about their local health services, and feel they have little influence on improving them. This raises questions about how we perceive non-White people. Systematic review data identified that negative implicit bias^5^ in healthcare providers towards people from ethnic minorities is linked to poor interactions, such as less respect and worse patient outcomes. Furthermore, racism (treating other people differently based on their ethnic origin) is associated with poorer physical and mental health.^6^

*‘If you don't like something, change it. If you can't change it, change your attitude*.’ Maya Angelou

### Teaching

Many GPs teach in clinics, in classroom settings, and more recently online via video calls. However, our understanding of race in medicine has been inadequate and, in most cases, absent. Knowledge and awareness of how this topic has both clinical and social relevance may change how many view racism today. The history of medicine and the unethical experiments supporting several medical advancements are not explicit on medical curricula. Marion J Sims, who is described as the *‘* *father of modern gynaecology* *’* conducted experimental research on enslaved Black women without anaesthesia between 1845 and 1849. The Tuskegee study^7^ performed in the US (1932–1972) explored the effects of untreated syphilis in Black men, and sought to justify continued non-treatment even once a recommended treatment became available. Black people were seen as suitable subjects of inhumane experiments in the name of medical advances. The legacy of colonisation and its impact on culture and medicine is unavailable to medical students unless they choose to study a history of medicine module, intercalated degree, or through independent study. Efforts to change the curriculum to acknowledge such a legacy are called ‘decolonisation’.^8^ One particular effort to change the medical curriculum recently is a book^9^ to help clinicians identify skin disease on Black and brown skin, which is not commonplace in current dermatological textbooks.

Race, as a social construct with no genetic basis,^10^ touches on vital concepts that inform the way we practise medicine, such as vulnerable populations, inequalities, societal power, ethics, and harmful stereotyping. The impact of ‘weathering’ or accelerated biological aging,^11^ the accumulated strain from microaggressions and compromises, as well as the ‘acculturative stresses’ ^12^ experienced by migrant communities adjusting to new lives as minorities overseas, has a powerful impact on health. Many diseases we consider having ‘racial’ elements, particularly in diabetes, mental health, and cardiovascular disease, may well be socially patterned. This is why we need to talk about the impact of race in the curricula. We may not be able to change the past, but we must learn from it.

*‘The true human story ... appears to be not of pure races rooted in one place for tens of thousands of years, but of constant mixing, with migration both one way and another ... Race, nationality and ethnicity are not what we imagine them to be when seen from the deep past. They are ephemeral, real only in as much as we have made them real by living in the cultures we do, with the politics we have*.’ Angela Saini

### Leading

Is it unsurprising that those who lead us struggle with solutions? Representation matters, and while momentum is gathering behind the Workforce Race Equality Standard (WRES) in monitoring and holding NHS bodies to account for their ethnic diversity, we are still far from achieving equality. Currently the WRES covers secondary care alone, and perhaps should be more expansive to include clinical commissioning groups (CCGs), academia, and social care. It is currently unclear what the composition of the primary care leadership is in terms of gender, ethnicity, and disability, with the last survey available from 2013.^13^ Despite this there are local initiatives starting to tackle this, such as the Leeds primary care BAME network^14,15^ to address racial leadership disparities and tackle health inequalities.

Diverse leadership boards are not powerful statements or virtue signalling. Diversity in management is economically efficient,^16^ and makes smarter and more innovative^17^ teams which could save lives at the clinical coalface. The implementation of the Rooney rule^18^ (interviewing candidates from a minority ethnic background) could ensure recruitment based on merit rather than ‘who you know’. How do we create communities of practice to incubate leaders from ethnic minorities? Have we created safe spaces and staff networks that facilitate this? Even for those boards which have diverse talent in leadership, the work must go on. Representation cannot be at the expense of inclusive, compassionate, and value-driven cultures. Far too often, BAME leaders have struggled to articulate their true self in unwelcoming environments, which can perpetuate the cycle of indifference if they are seen as ‘ineffective’.

*‘Anti-racist practice cannot be grown if you are unwilling to hear and accept feedback from others, especially BAME people. Buying into the narrative of “racism is perpetrated by bad people, I am a good person and therefore not racist” is both naïve and unhelpful and stifles useful debate about creating race equality*.’ Tracie Joliff

### Relaxing

Greater awareness and understanding is key, both at work and in our home lives. Anybody who has tried to buy a toy or a book with a non-White central character,^19^ or buy a plaster in an appropriate colour for different skin tones, will know that it can be challenging. When we turn electric lights in our home, do we realise that it was Lewis Latimer, a Black inventor, who improved Thomas Edison’s initial invention by means of a carbon rather than paper filament (as Edison had used)? Even examination of football commentary showed that darker skin players were 7 and 3 times^20^ more likely to be commended on their power and speed, respectively, over work ethic. When we go home how do we ensure that, within our personal lives, we strive to recognise and resolve issues relating to lack of diversity and racism? Do we challenge casually racist or culturally ignorant comments made by our family and friends? Sometimes it is harder to challenge those we love, but this is important.

Learning at home has a huge impact on children and their social development. Children are not born with intrinsically racist views; these are learnt from experience and environment. Anybody familiar with the ‘Doll Test’, where ‘racial identification and preference’ was explored with children, will be aware that ideas about identity and preference relating to race can form at a very early age, even in children as young as 5. Challenging negative and incorrect stereotypes helps children to understand issues which may not directly affect them, and helps to make them more aware of the problems that BAME populations face. Resources available to help start conversations include episodes from BBC Bitesize^21^ and Race and Health^22^ which provide readily accessible information for families to share together.

*‘You are personally responsible for becoming more ethical than the society you grew up in.’* Eliezer Yudkowsky

## Conclusion

Some of us who have experienced racism now see race everywhere; much like holding a hammer, everything looks like nails. It is comfortable to ignore racial disparities and racism. Perhaps if we do not see them, they do not exist. But, as Angela Davis said ‘*it is* *not enough* *to be non-racist* *;* *we must be anti-racist*’. Ijeoma Oluo described anti-racism *as ‘the commitment to fight racism wherever you find it, including* *in yourself’*. This is a continuous process and involves educating ourselves through any means that are available to us: see Race & Health,^22^ for example. Acknowledgement of and countering our own prejudices will be uncomfortable. We must choose discomfort. Choose to make the change.

*‘Not everything that is faced can be changed, but nothing can be changed until it is faced*.’ James Baldwin

## References

[1] Dhairyawan R (2020). Evaluating Values: BMJ Leader. https://blogs.bmj.com/bmjleader/2020/06/15/evaluating-values-by-rageshri-dhairyawan/.

[2] Draper ES, Gallimore ID, Smith LK (2019). MBRRACE-UK Perinatal Mortality Surveillance Report, UK Perinatal Deaths for Births from January to December 2017. Leicester: The Infant Mortality and Morbidity Studies, Department of Health Sciences, University of Leicester. https://www.npeu.ox.ac.uk/assets/downloads/mbrrace-uk/reports/MBRRACE-UK%20Perinatal%20Mortality%20Surveillance%20Report%20for%20Births%20in%202017%20-%20FINAL%20Revised.pdf.

[3] Tarlov AR (1999). Public policy frameworks for improving population health. Ann N Y Acad Sci.

[4] Lakhani M (2008). No Patient Left Behind: how can we ensure world class primary care for black and minority ethnic people? : Department of Health. http://www.em-online.com/download/medical_article/36782_DH_084973.pdf.

[5] Hall WJ, Chapman MV, Lee KM (2015). Implicit racial/ethnic bias among health care professionals and its influence on health care outcomes: a systematic review. Am J Public Health.

[6] Priest N, Paradies Y, Trenerry B (2013). A systematic review of studies examining the relationship between reported racism and health and wellbeing for children and young people. Soc Sci Med.

[7] Centers for Disease Control and Prevention (2020). US Public Health Service Syphilis Study at Tuskegee: The Tuskegee Timeline. https://www.cdc.gov/tuskegee/timeline.htm.

[8] Lokugamage AU, Ahillan T, Pathberiya SDC (2020). Decolonising ideas of healing in medical education. J Med Ethics.

[9] Mukwende M, Tamony P, Turner M (2020). Mind The Gap: A Handbook of Clinical Signs in Black and Brown skin. https://www.blackandbrownskin.co.uk/mindthegap.

[10] Witherspoon DJ, Wooding S, Rogers AR (2007). Genetic similarities within and between human populations. Genetics.

[11] Geronimus AT, Hicken MT, Pearson JA (2010). Do US Black women experience stress-related accelerated biological aging?. Hum Nat.

[12] Sam DL, Berry JW (2010). Acculturation: when individuals and groups of different cultural backgrounds meet. Perspect Psychol Sci.

[13] NHS England (2013). Clinical Commissioning Group Workforce Equality and Diversity Profile. NHS England [Publications Gateway Reference 00835]. https://www.england.nhs.uk/wp-content/uploads/2013/12/nationaled_surveys_V8-2013-12-06.pdf.

[14] Sattar M (2020). Primary care BAME network: Leeds Clinical Commissioning Group. https://www.leedsccg.nhs.uk/about/covid-19-primary-care/resources-for-professionals/primary-care-bame-network/.

[15] Richardson J (2020). How *The BMJ*'s racism special inspired a Leeds GP to set up an ethnic minority staff network. BMJ.

[16] McGregor-Smith R (2017). Race in the workplace: The McGregor-Smith Review: Department for Business, Energy & Industrial Strategy. UK Government. https://www.gov.uk/government/publications/race-in-the-workplace-the-mcgregor-smith-review.

[17] Rock D, Grant H (2016). Why diverse teams are smarter. *Harvard Business Review* [online]. https://hbr.org/2016/11/why-diverse-teams-are-smarter.

[18] Kar PS (2020). To tackle racism, the NHS needs policies with teeth: BMJ. https://www.bmj.com/content/369/bmj.m2583.

[19] Bold MR (2019). Representation of people of colour among children’s book authors and illustrators: BookTrust. https://www.booktrust.org.uk/globalassets/resources/represents/booktrust-represents-diversity-childrens-authors-illustrators-report.pdf.

[20] McLoughlin D (21 Jul 2020). Racial bias in football commentary (study). *RunRepeat* [online]. https://runrepeat.com/racial-bias-study-soccer.

[21] (2020). How to talk to your children about Black Lives Matter: BBC Bitesize. https://www.bbc.co.uk/programmes/p08gyw71.

[22] Race, Xenophobia, Discrimination & Health (2020). Race & Health Resource Hub. https://raceandhealth.org/resources.html.

